# Investments in Community Building Among Nonprofit Hospital Organizations in the United States

**DOI:** 10.1001/jamanetworkopen.2020.21898

**Published:** 2020-10-23

**Authors:** Kevin Chen, Katherine L. Chen, Leo Lopez

**Affiliations:** 1 National Clinician Scholars Program, Yale University School of Medicine, New Haven, Connecticut; 2 National Clinician Scholars Program, UCLA (University of California, Los Angeles), Los Angeles

## Abstract

This cross-sectional analysis of a national sample of nonprofit, acute care hospital organizations assesses how these organizations distribute spending for community-building activities among the 9 domains allowed by the Internal Revenue Service.

## Introduction

Nonprofit hospital organizations in the United States receive federal tax exemption with the expectation that they act to understand and address the health care needs of the communities they serve. The Internal Revenue Service requires that they report expenditures toward these activities on a form called the Schedule H. Spending is classified as community benefits or community building.^1^ Community benefits largely describe unreimbursed and subsidized health care services.^2,3^ Community building describes activities to “protect or improve the community's health or safety” not otherwise reported as community benefits.^1^ Hospitals must report community benefits for tax exemption. For community-building activities to count toward tax exemption, organizations must supply additional documentation to reclassify them as community benefits.^1^

The Internal Revenue Service allows hospital organizations to report community building in 9 domains—such as physical improvements and housing, economic development, and environmental improvements—related to social determinants of health.^1^ According to estimates reported in some studies, nonprofit hospital organizations spend about 0.1% of operating expenses on community building,^2,3^ but little is known about organizations’ contributions in each domain.

This study aimed to describe the distribution of spending in community building among the 9 domains by nonprofit hospital organizations.

## Methods

We conducted a cross-sectional analysis of a national sample of nonprofit, acute care hospital organizations (entities operating 1 or more hospital facilities) for fiscal year 2016. Because this study did not involve research with human subjects, the Yale University Institutional Review Board indicated that it did not require institutional review board review or approval. We followed the Strengthening the Reporting of Observational Studies in Epidemiology (STROBE) reporting guidelines for cross-sectional studies.

We used data from the Community Benefit Insight Hospital Data Set.^4^ Community Benefit Insight integrates information from Schedule Hs, American Hospital Association annual surveys, and Centers for Medicare and Medicaid Services cost reports and contains a nearly complete (>85%) sample of nonprofit hospital organization filings for fiscal year 2016.^3,4^ We used Stata SE, version 15 (StataCorp) to generate descriptive statistics of sample characteristics and expenditures in each community-building domain.

## Results

We identified 2102 unique Schedule H filings, representing 2903 hospitals from 2100 hospital organizations (Table 1). More than half of the hospital organizations (1140 [54.3%]) reported any spending on community building. Of those, community building accounted for a median of 0.04% (interquartile range, 0.009%-0.1%) of total operating expenses. Overall, hospital organizations contributed $434 million toward community building. Together, community support and workforce development accounted for 53.7% of all community-building investments, with the remaining 46.3% split among the other 7 domains (Table 2).

**Table 1.** Sample Characteristics

Characteristic	No. (%)
**Nonprofit hospital organizations (n = 2100)**^a^	
No. of hospitals in organization^b^	
1	1845 (87.9)
2-3	176 (8.4)
4-12	67 (3.2)
12-34	12 (0.6)
Total operating expenses, million, $^c^	
<100	954 (45.4)
100-299	513 (24.4)
≥300	633 (30.1)
**Nonprofit hospitals (n = 2903)**	
No. of beds	
<100	1291 (44.5)
100-299	932 (32.1)
≥300	680 (23.4)
Teaching^d^	921 (31.7)
Rural	998 (34.4)
Region^e^	
Northeast	498 (17.2)
Midwest	980 (33.8)
South	899 (31.0)
West	526 (18.1)

^a^ Our sample included 2100 unique hospital organizations filing 2102 Schedule Hs; 2 hospital organizations filed separate Schedule Hs for individual hospitals within their organizations.

^b^ The median count was 1 hospital, 75th percentile was 3, 90th percentile was 12, and maximum was 34.

^c^ Sum of percentages may not be exactly 100 because of rounding.

^d^ As determined by membership in the Council of Teaching Hospitals.

^e^ As defined by the US Census Bureau’s geographic regions; sum of percentages may not be exactly 100 because of rounding.

**Table 2.** Community-Building Spending in Fiscal Year 2016 Among 2100 Nonprofit Hospital Organizations

Community-building domain	Total spending in community-building domain (all hospital organizations), $	Hospital organizations with any community-building spending in domain, No. (%)	Median (IQR)^a^	
			Community-building spending amount, $	Percentage of total community-building spending amount in domain
All domains	434 157 152	1140 (54.3)	63 572 (12 246-242 138)	NA
Community support^b^	109 641 208	727 (34.6)	14 424 (3004-65 825)	35.5 (10.3-81.3)
Workforce development^c^	123 399 544	567 (27.0)	25 849 (3862-163 077)	42.2 (7.4-88.4)
Community health improvement advocacy^d^	66 242 235	447 (21.3)	12 839 (2500-52 851)	18.7 (3.9-65.0)
Coalition building^e^	23 294 527	423 (20.1)	7540 (2000-26 202)	13.1 (3.6-42.1)
Economic development^f^	32 003 158	377 (17.9)	5684 (1607-20 000)	10.2 (2.8-29.2)
Leadership development and training for community members^g^	5 461 979	199 (9.5)	4191 (1080-14 858)	5.0 (1.0-17.8)
Physical improvements and housing^h^	36 612 366	186 (8.8)	9916 (1571-48 187)	7.5 (1.5-32.3)
Other^i^	31 702 399	163 (7.8)	39 076 (5218-144 083)	39.8 (10.3-94.4)
Environmental improvements^j^	5 853 488	153 (7.3)	3077 (500-26 691)	4.7 (0.9-20.7)

Abbreviation: NA, not applicable.

^a^ Of hospitals that had any community building spending in a given domain.

^b^ Community support includes mentoring programs for vulnerable neighborhoods, violence prevention programs, and disaster preparedness activities.^1^

^c^ Workforce development includes training and recruitment of health care professionals needed in the community.^1^

^d^ Community health improvement advocacy includes advocacy in support of policies and programs related to public health, health services, and social determinants of health.^1^

^e^ Coalition building includes collaborations with the community to improve health and safety.^1^

^f^ Economic development includes job creation and small business support in economically vulnerable neighborhoods.^1^

^g^ Leadership development and training for community members include training community residents in leadership domains such as conflict resolution and language skills.^1^

^h^ Physical improvements and housing include providing and rehabilitating housing for vulnerable populations and investing in parks to support physical activity.^1^

^i^ Other describes activities that support the health and safety of the community and are not otherwise included in the other community building domains.^1^

^j^ Environmental improvements include efforts to reduce or remove environmental hazards in the community such as pollution and toxic waste.^1^

## Discussion

Most hospital organizations reported spending toward community building in fiscal year 2016. More than half of spending focused on community support and workforce development, consistent with findings from a study of hospitals in New York state.^5^ To our knowledge, this is the first study to detail community-building investments by domain in a national sample. Further investigation is needed to understand how and why hospitals decide to allocate money toward specific community-building domains and whether these investments are effective in improving community health.

This study has some limitations. Because the Internal Revenue Service does not require spending and reporting in community building, hospitals may participate in community building without reporting it on the Schedule H. In addition, because Schedule H data are generally reported by hospital organizations, attribution of investments to individual facilities within an organization is not possible.

Nonetheless, the Schedule H can be a tool for public accountability and a starting point for evaluating how hospitals engage with social determinants of health. Additional reform to the classification and incentivization of community building reporting may help enhance the social contract between nonprofit hospital organizations and their communities. Examples of such reforms include identifying clearly defined, evidence-based community-building activities that would count toward tax exemption and reducing documentation requirements for receiving credit for community building.^6^
